# Efficient OLEDs Based on Slot-Die-Coated Multicomponent Emissive Layer

**DOI:** 10.3390/polym14163363

**Published:** 2022-08-17

**Authors:** Ewelina Witkowska, Ireneusz Glowacki, Tung-Huei Ke, Pawel Malinowski, Paul Heremans

**Affiliations:** 1 Department of Molecular Physics, Lodz University of Technology, Zeromskiego 116, 90-924 Lodz, Poland; 2 Interuniversity Microelectronics Centre (IMEC), Kapeldreef 75, B-3001 Leuven, Belgium

**Keywords:** OLEDs, printed electronics, printed organic device, slot-die coating, TADF assistant dopant

## Abstract

The optimization of multicomponent emissive layer (EML) deposition by slot-die coating for organic light-emitting diodes (OLEDs) is presented. In the investigated EMLs, the yellow-green iridium complex (Ir) was doped in two types of host: a commonly used mixture of poly(*N*-vinylcarbazole) (PVK) with oxadiazole derivative (PBD) or PVK with thermally activated delayed fluorescence-assisted dopant (10-(4-(4,6-diphenyl-1,3,5-triazin-2-yl)phenyl)-10H-spiro[acridine-9,9′-fluorene], SpiroAC-TRZ). In this article, OLEDs with EML prepared in air by slot-die coating, facilitating industrial manufacturing, are confronted with those with spin-coated EML in nitrogen. OLEDs based on PVK:PBD + 2 wt.% Ir-dopant exhibit comparable performance: ~13 cd A^−1^, regardless of the used method. The highest current efficiency (21 cd A^−1^) is shown by OLEDs based on spin-coated PVK with 25 wt.% SpiroAC-TRZ and 2 wt.% Ir-dopant. It is three times higher than the efficiency of OLEDs with slot-die-coated EML in air. The performance reduction, connected with the adverse oxygen effect on the energy transfer from TADF to emitter molecules, is minimized by the rapid EML annealing in a nitrogen atmosphere. This post-treatment causes more than a doubling of the OLED efficiency, from 7 cd A^−1^ to over 15 cd A^−1^. Such an approach may be easily implemented in other printing techniques and result in a yield enhancement.

## 1. Introduction

Organic light-emitting diodes (OLEDs) are an increasingly popular technology for large-area flat panel displays and lighting applications since they offer promising features, including low power consumption, high color purity, a wide viewing angle, the possibility of manufacturing in a versatile form and design, and the ability to be flexible [1,2]. OLEDs can be fabricated using two distinct methods: by thermal vacuum evaporation or by solution processes [3]. To date, commercially available OLEDs are obtained mainly by vacuum processes. They allow precise layer thickness control and support high device efficiency [4]. However, due to the high power consumption of the vacuum deposition system, the significant waste of materials, and limitations concerning large-area fabrication, the interests of both the academic and industrial communities are shifting towards OLEDs manufactured by solution process techniques [5,6]. Solution techniques allow the fabrication of large-area, flexible, and thin devices [7]; at the same time, they are cost saving and could be relatively environmentally friendly. However, the fabrication of thin and homogeneous multilayer structures from solution is challenging due to possible destruction of the previously deposited layers by subsequent coating from a solution [8,9]. Due to the material solubility problem, the control of the thin-film morphology during printing/coating processes is difficult, and defects are quite often observed [10,11,12]. The aforementioned reasons lead to limitations in terms of the efficiency, lifetime, and reproducibility; thus, vacuum-processed OLEDs still exhibit better performance [13,14]. Consequently, the adoption of solution techniques is still laggard in mass production [15].

Wet processes for the fabrication of OLEDs could be divided into methods with or without in situ patterning. The coating method without in situ patterning is characterized by film deposition on the entire area of the device, and separate pixels generally cannot be obtained without additional patterning steps. Thus, such processes might be mostly applied for the tests and for OLED lighting. In the case of OLED displays, a full-color device can be obtained in different ways, such as filtering white light and patterning pixels (using three basic colors: blue, green, and red) by applying efficient dyes, which adjusts colors [3]. The most promising solution is the fabrication of separated RGB (red, green, and blue) pixels due to more efficient utilization of the generated light [16]. Therefore, in situ pattering methods, such as inkjet printing, aerosol-jet printing, and nozzle printing, seem to be more suitable in this case [17]. Nevertheless, the introduction of, e.g., photolithographic patterning can overcome large-area-coating limitations in the methods without in situ patterning [18,19].

The most widely applied example of this type of method is the spin-coating technique [20,21,22,23]. It is characterized by good reproducibility of uniform thin-layer formation. Other coating processes without fine patterning are spray coating, blade coating, dip coating, slit-nozzle coating [3], and slot-die coating [24]. The latter process could be easily applied in mass production, even in roll-to-roll applications [25]. In such a system, thin (a few nanometers) and relatively thick layers (a few micrometers) might be obtained depending on the solution viscosity and other coating conditions [26]. However, such a system is much more difficult to optimize due to the large number of process parameters [27,28]. Nevertheless, among many coating techniques, the slot-die technique is widely used to fabricate optoelectronic devices such as organic photovoltaics (OPVs) [29,30,31,32], sensors [33,34,35], organic thin-film transistors (OTFTs) [36,37,38], and OLEDs [25,39,40,41,42,43,44] in both single- [45] and multi-layer configurations [46]. Emissive layers (EMLs), poly(3,4—ethylenedioxythiophene) and poly(styrene sulfonate) mixture (PEDOT:PSS) [43,45,47,48], and other supportive layers [48,49,50] have been produced through this technique. The fabrication of single- and multicomponent EMLs has been investigated [41].

As previously mentioned, in solution-processed multilayer OLEDs, the challenge is to avoid the re-dissolution of previously deposited layers when subsequent solutions are applied. Therefore, the selection of an appropriate solvent is fundamental. With the slot-die-coating technique, this process is facilitated by the fact that the solvent selection is not as drastically restricted as in the case of print heads. In addition, there is no need to meet such stringent ink rheological parameters to create a droplet, as is the case with inkjet printing applications [51].

An additional essential benefit of the slot-die technique is that it is significantly faster in comparison to, for instance, inkjet printing, reducing the negative impact of the air environment. Slot-die coating ensures reduced oxygen contact with the deposited solution. Additionally, the fluid is coated on the substrate and air contact is limited to the substrate surface and the surface area of the created layer. In contrast, inkjet printing results in higher exposure to oxygen, which can affect the material’s functionality. In inkjet printing, a droplet of a solution is surrounded by air before it reaches the sample surface. In addition, it must be taken into account that the droplet hits the substrate at a high velocity, while at the same time the solvent evaporates quickly. Previous research demonstrated the creation of a small bubble inside the droplet, close to the substrate [52,53]. The air entrapment model explains the size of the bubble in the early stages of impact [52]. As Mehdi-Nejad and coworkers explained, when the droplet approaches another surface, air in the gap between the substrate and the droplet is forced out. Increased air pressure below a droplet creates a dimple in its surface in which air is trapped [53]. Therefore, more oxygen molecules may be pressed into the bulk of the formed layer during the deposition by inkjet printing. Consequently, there might be more oxygen molecules trapped in the volume of a relatively thick layer (~70 nm). Their removal, by annealing in an inert atmosphere, is challenging. The ability to release adsorbed air only from the part close to the surface of slot-die-coated layer seems to be easier.

Taking into account all the reasons described above, slot-die coating was selected for this study as a technique with a minimal negative impact of ambient conditions and simultaneous ease of application to multilayer OLED systems.

The first part of this manuscript is focused on the optimization of slot-die-coating process parameters consisting of the substrate speed, solution flow rate, temperature, and solvent adjustment. In the second part, we have performed studies on multicomponent EML, whose complex compositions make it more demanding to achieve proper layer quality in comparison to single-component layer types. Two EML combinations, prepared previously by the spin-coating method, were selected for further investigation. In the first, an emissive layer was based on a polymer host–guest system; in the second one, a polymer host–guest system was doped with thermally activated delayed fluorescence (TADF) material. These two systems employed in OLEDs were compared for spin and slot-die coating. It is necessary to underline that, to our knowledge, the slot-die coating of the polymer blend with TADF assistance material has not yet been reported. It is worth checking whether it is beneficial to employ the slot-die-coating method for the different emissive systems in a similar way, with analogous efficiency. The studies indicate that this method is appropriate for complex EML compositions as well the system enriched with TADF-type materials. The third part of the article is related to an analysis of oxygen’s impact on the performance of OLEDs with the polymer-TADF-Ir-system-based EML. The post-treatment approach for the deposited layer, in order to obtain a relatively efficient multilayer OLED, was additionally established.

## 2. Materials and Methods

### 2.1. Slot-Die Coating Technique

**Figure 1:**
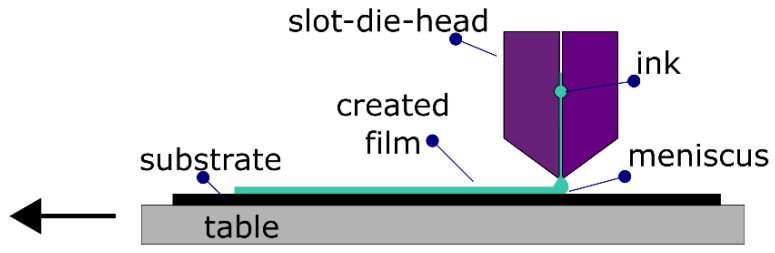
Scheme of slot-die-coating method.

A simplified scheme of the slot-die-coating system is presented in Figure 1. Generally, the system comprises the following main parts: an ink reservoir (e.g., glass syringe), pumping system, slot-die head, and a substrate table or roller. The ink is supplied from the reservoir into the slot-die head with a controlled pumping speed. The slot-die head consists of an ink distributor chamber and a nozzle. The distributor chamber helps to obtain even ink distribution within the slot-die head and appropriate ink flow to the opening of the head. The nozzle lip defines the coating width and can be used to establish a stripe pattern [24]. The substrate holder may be a roller or a flat table and may provide heating options. The ink stored in the container is pumped with a preset pumping rate into the slot-die head and comes out from the slot-die outlet to form a meniscus between the slot-die nozzle and the substrate. The meniscus shifts during the movement of the substrate to create the film.

The quality and thickness of the thin film depend strongly on the coating settings, slot-die type, process variables, and ink composition (solvent, solution concentration). Slot-die-coating optimization consists of the adjustment of several parameters, including: the substrate temperature, conditions of meniscus creation, substrate speed, and solution dispensing rate [40,54]. It is critical to create the proper meniscus of the solution between the nozzle and the substrate [28]. This is a very important step since it, for example, determines whether the whole area will be covered or, on the contrary, whether the material will overspread on the substrate. Furthermore, the applied system allows the nozzle angle to be changed. Additionally, the solution flow speed and substrate speed need to be adjusted to receive the same layer thickness on the entire length of the sample. Moreover, the substrate temperature, solution concentration, and selected solvent could influence the thickness and uniformity of the formed film. The slot-die-coating system could be adapted to work in an inert atmosphere, as is the case with spin-coating. Nevertheless, due to its scalability and potential to be applied in the industry, tests were performed in air.

### 2.2. Slot-Die Coating Optimization

The slot-die-coating process was optimized for the deposition of an emissive layer on indium tin oxide (ITO) glass substrates (3 × 3 cm) previously coated with PEDOT:PSS. Two types of host–guest EMLs were investigated. One was based on the mixture of poly(*N*—vinylcarbazole) (PVK) with a 30 wt.% oxadiazole derivative (PBD) doped with 2 wt.% of phosphorescent [bis(benzo[*h*]quinolinato-*N*,*C*^10^′){4-((1-naphtyl)imino)-pent-2-en-2-olato-*N,O*} iridium(III)] complex (Ir). In the second case, PBD was replaced by 25 wt.% of 10-(4-(4,6-diphenyl-1,3,5-triazin-2-yl)phenyl)-10H-spiro[acridine-9,9′-fluorene] (SpiroAC-TRZ), which was applied as TADF assistance dopant.

As was described above, slot-die-coating process optimization involves the adjustment of several parameters. To obtain a 75 nm emissive layer, the following parameters were selected: nozzle under angle: ~35°, substrate temperature: 40 °C, distance between the nozzle and the substrate: 50 μm. To create the meniscus, 20 μL was pumped with a solution flow rate of 50 μL min^−1^, whereas, during the deposition, the solution flow was reduced to 10 μL min^−1^ and the substrate moved at a speed of 7.0 mm s^−1^ or 10 mm s^−1^. The PVK:PBD + Ir emissive layer was deposited from a mixture of anisole and chlorobenzene (1:1 by volume) at 21 mg mL^−1^, whereas the PVK:SpiroAC-TRZ + Ir solution was applied at 18 mg mL^−1^. The layer properties were controlled by an optical microscope, profilometer, or atomic force microscope (AFM). The layers were checked under a polarizing microscope to control the phase separation after annealing. In addition, no differences were observed in absorption and emission spectra.

### 2.3. OLEDs Preparation

The OLEDs were prepared on ITO glass substrates (3 × 3 cm) by the spin- and slot-die-coating techniques assisted by thermal vacuum deposition. Primarily, in the air environment, PEDOT:PSS was spin coated as a hole injection layer on the ITO anode. Before EML deposition, the PEDOT:PSS layer was baked at 120 °C for 10 min to remove any remaining water. Second, the EML, composed of PVK with PBD or SpiroAC-TRZ doped with Ir complex, was spin coated (in a glovebox) or slot-die coated (in air conditions). To remove the residual amounts of solvent, the deposited EMLs were baked at 90 °C for 30 min under a nitrogen atmosphere and then kept in vacuum overnight. Finally, the other layers were patterned through the shadow masks by thermal vacuum deposition in order to prepare twelve diodes on one substrate. 1,3,5-tris(*N*-phen-ylbenzimidazol-2-yl)-benzene (TPBi) or 4,7-diphenyl-1,10-phenanthroline (Bphen) were applied as an electron transport layer (ETL) and (8-quinolinolato)lithium (Liq) as an electron injection layer. The device was completed by a silver cathode (Ag). In summary, the full OLED structure can be described as follows: ITO/PEDOT:PSS (20 nm)/EML (75 nm)/ETL (20 nm or 40 nm)/Liq (2 nm)/Ag (100 nm). The device electroluminescence (EL) and current density–voltage–luminance (*J-V-L*) characteristics were collected via a Keithley 237 source-measurement unit paired with a Minolta CS-2000a camera.

## 3. Results and Discussion

### 3.1. Optimization of Slot-Die-Coating Process Parameters

Process parameters were optimized for layer composition of the matrix mixture of PVK:PBD with the addition of 2 wt.% of iridium complex (Ir). The applied concentration (2 wt.%) of the phosphorescent emitter in the emissive layer was based on our previous studies [55].

The preparation of solution-processed OLEDs is challenging as the coating solvent of an organic layer can dissolve the underlayer and layers become mixed. Therefore, the selection of an appropriate solvent that enables uniform coating of the layer and simultaneously does not dissolve or swell the underlayer is crucial. Furthermore, it is known that the thickness and layer quality are also influenced by the surface tension arising between the solution and substrate surface. For this reason, optimization of the EML slot-die coating was carried out directly on substrates with a previously spin-coated PEDOT:PSS film (~20 nm), which was introduced as the hole injection layer (HIL). It should be pointed out that in our studies, the thickness of the EML was monitored by measuring the thickness in relation to the ITO glass substrate (including the PEDOT:PSS layer). The HIL/EML interface studies have not been performed. Therefore, it is not known to what extent the EML slot-die coating dissolves or swells the surface of the PEDOT:PSS, which can result in a change in its nominal thickness (theoretically 20 nm). Nonetheless, for different layer depositions, orthogonal solvents were applied, and the minimization of such effects is presumed.

As process settings are dependent on each other, their selection is not straightforward, and in turn, high layer smoothness is required for OLED application. After selecting the appropriate conditions for meniscus formation (the angle and distance of the nozzle from the substrate and the nozzle filling rate) and the solution (solvent, concentration), it can be assumed that the temperature and flow rate of the solution as well as the speed of the moving substrate are the main parameters influencing the film morphology [50]. Therefore, the effect of these parameters on the film formation was investigated more thoroughly.

The first tests were made for chlorobenzene because it was used in a previously optimized spin-coating process [55]. After the EML coating, all samples were baked under a nitrogen atmosphere to remove the residual amounts of the solvent and stabilize the structure of the film. Thickness as a function of most crucial process parameters (substrate speed, solution flow rate, and temperature) is presented in Figure S1. Nonetheless, the film quality was unsatisfactory due to the high roughness of the layer. A large change in thickness and its irregularity as a consequence of the varying substrate speed (in the range of 0.2–0.5 mm s^−1^) and the solution flow rate (in the range of 5–10 µL min^−1^) were obtained. As can be seen in Figure S2, the layer was folded and stripes across the substrate movement were observed. Visible lines across the substrates might have been caused by a too slow evaporation rate, connected with improper method parameters and solvent properties. Further substrate speed increment and temperature reduction resulted only in layer thinning, without significant quality improvement. It is known that, in parallel with the deposition parameters, the choice of the solvent itself is important, due to the possibility of change in the surface tension. Therefore, in the next step, different solvents were investigated (Figure S4).

**Figure 2:**
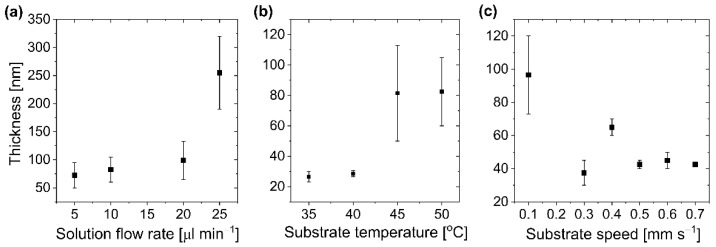
Thickness (including PEDOT:PSS) of slot-die-coated EMLs (PVK:PBD + 2 wt.% Ir) deposited at different process parameters from anisole. (**a**) Thickness vs. solution flow rate (nozzle-substrate distance: 100 μm, temperature: 50 °C, substrate speed: 0.3 mm s^−1^); (**b**) thickness vs. substrate temperature (nozzle–substrate distance: 100 μm, flow rate: 10 μL min^−1^, substrate speed: 0.3 mm s^−1^); (**c**) Thickness vs. substrate speed (nozzle–substrate distance: 100 μm, temperature: 50 °C, flow rate: 5 μL min^−^^1^).

Detailed process condition optimization was performed for anisole, as a relatively safe type of solvent for the user (non-mutagenic, non-carcinogenic) with a high boiling point (Figure 2 and Figure S3). The first tests indicated that the distance between the substrate and the nozzle should not be greater than 100 μm to obtain an HIL/EML thicker than 40 nm. Going forward, as shown in Figure 2a, an increase in the flow rate of solution above 20 μL min^−1^ results in an overflow, and as a consequence, thick ( >100 nm) and extremely non-uniform layers are obtained. Therefore, solution flow in a range of 5–10 μL min^−1^ seems to be the most appropriate for the setup applied. Furthermore, the temperature dependence (Figure 2b) shows that keeping the substrate temperature in a range of 35–40 °C ensures smoothness of the layer, whereas an increase above 45 °C results in huge inhomogeneity in the layer thickness. The substrate speed was examined in the range of 0.1–0.7 mm s^−1^ and revealed that the best results can be obtained in the range of 0.5–0.7 mm s^−1^ (Figure 2c). Additionally, microscope images showed good quality of the layers received from anisole with optimized process parameters (Figure S3). However, when the layer morphology was satisfactory, the thickness of the emissive layer together with PEDOT: PSS was only ~40 nm, which is not enough to ensure its stable work. Inter alia breakthroughs during the device operation can be observed in the case of thin EML (~20 nm), applied in the simple OLED structure. They can be overcome by the introduction of a stack of several supportive layers. Nevertheless, such an approach is extremely difficult to implement in solution processing. Consequently, the deposition conditions were further optimized to receive an EML thickness above 70 nm.

**Figure 3:**
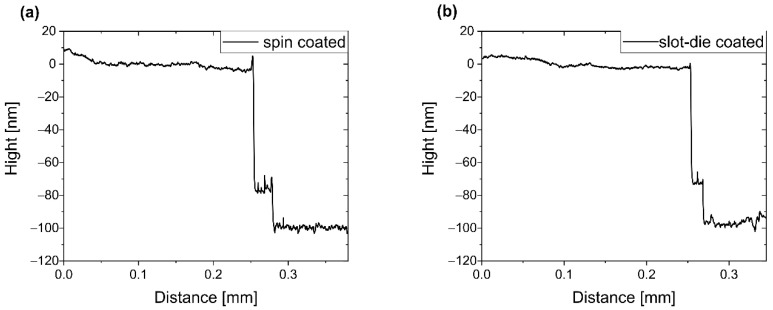
Profilometry diagrams of emissive layer (PVK:PBD + Ir) deposited from anisole:chlorobenzene mixture, by spin coating (**a**) and slot-die coating (**b**). Emissive layer deposited on PEDOT:PSS/ITO. The first downcast refers to PEDOT:PSS layer, and the second one to the ITO anode.

Different solvents were introduced into the studies (Figure S4). Based on results previously obtained for chlorobenzene and anisole, tests were also conducted for their mixture (1:1 by volume). To receive a 75 nm emissive layer, the following parameters were chosen: solution concentration: 21 mg mL^−1^, nozzle angle: ~35°, substrate temperature: 40 °C, distance between the nozzle and the substrate: 50 µm. To create the meniscus, 20 µL was pumped with a solution flow speed of 50 µL min^−1^, whereas during the deposition, the solution flow rate was reduced to 10 µL min^−1^ and the substrate moved at 7.0 mm s^−1^. The good quality of the obtained layer was proved by profilometer measurements and optical microscope images (Figure 3b and Figure S5). No aggregates were detected by both techniques. Additionally, the layer was not folded, which is a common indication of improper process adjustment. Furthermore, no differences were observed between the EMLs obtained by slot-die and spin-coating (see Figure 3 and compare Figure S5 with Figure S6).

### 3.2. OLED Results for Polymer Host–Guest System

**Figure 4:**
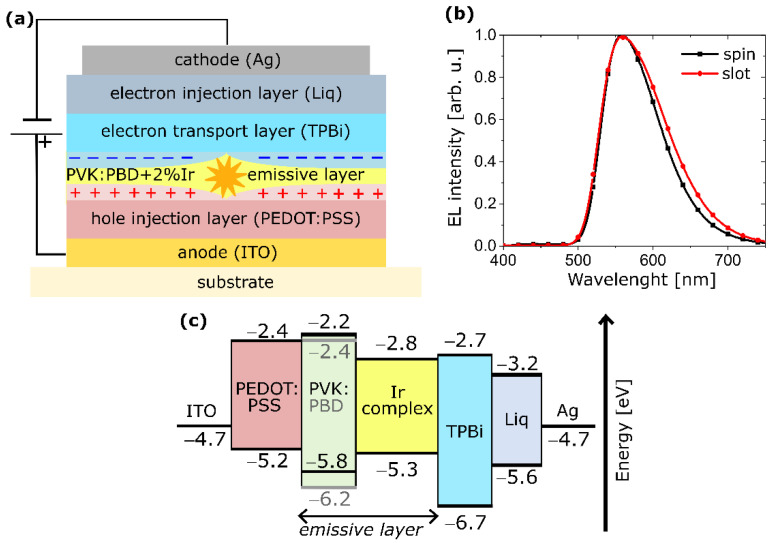
(**a**) Scheme of applied OLED structure; (**b**) normalized EL spectra of devices with emissive layers (PVK:PBD + 2 wt.% Ir) prepared by means of spin- or slot-die coating techniques; (**c**) energy level diagram for components applied in the OLED (ITO [56], PEDOT [57], emissive layer components [55], TPBi [58], Liq [59], Ag [60]).

**Figure 5:**
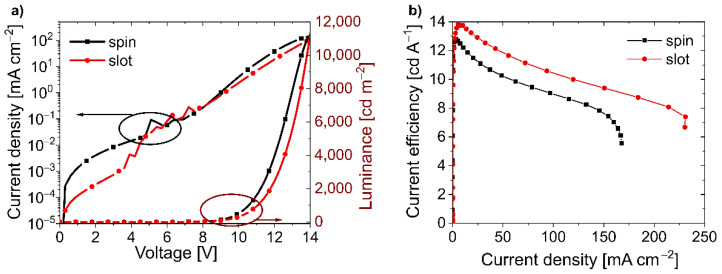
Working parameters of OLEDs with emissive layers prepared by spin- and slot-die coating techniques. Device structure: ITO/PEDOT:PSS(20 nm)/PVK:PBD + 2 wt.% Ir(75 nm)/TPBi(20 nm)/Liq (2 nm)/Ag (100 nm). (**a**) Current density–voltage characteristics (left) and luminance–voltage characteristics (right); (**b**) current efficiency vs. current density.

In the case of the host–guest system based on a polymer matrix and phosphorescent emitter (Ir), the device structure was as follows: ITO/PEDOT:PSS (20 nm)/PVK:PBD + 2 wt.% Ir (75 nm)/TPBi (20 nm)/Liq (2 nm)/ Ag (100 nm), and is depicted in Figure 4a. OLEDs with EML prepared in air by slot-die coating are confronted with those with spin-coated EML in nitrogen. After coating, spin and slot-die EMLs were annealed thermally in a nitrogen atmosphere. The results for OLEDs are presented in Figure 4b and Figure 5.

An electroluminescence (EL) spectrum (in 480–750 nm range) with maximum ca. 560 nm is associated with the Ir complex molecules [55]. However, for the emissive layer prepared by the slot-die coating, the EL band is slightly wider, with a long-wavelength arm formed (Figure 4b). Such spectrum broadening in the red region, might be related to the early stage of the emitter aggregate formation [61,62,63]. A longer solvent evaporation stage, in the case of the slot-die-coating method, could induce the creation of a small number of emitter aggregates. Nevertheless, studies under a polarizing microscope did not indicate the occurrence of emitter molecular aggregation. On the contrary to the slot-die method, the process of solid-layer creation in the spin-coating might be too fast to allow molecule aggregation.

As can be seen in Figure 5, fabricated OLEDs are characterized by a luminance (*L*) above 10,000 cd m^−2^ (at 14 V) and maximal current efficiency (*η*) between 13 and 14 cd A^−1^. Nonetheless, visible variability of current values on current density (*J*)–voltage (*V*) characteristics (left red *J*–*V* curve in Figure 5a) might be related to some instabilities of the device prepared by slot-die coating. That could be expected considering that the emissive layer consisting of a phosphorescent emitter was deposited in the air environment [64,65]. However, the current density is slightly lower and the current efficiency a little higher compared to devices with EML fabricated by the spin-coating method under inert conditions (see Figure 5). Concerning the power efficiency, its estimated maximal value is higher for spin-coated OLEDs. It is due to the same current achieved at lower voltage. However, the decrease in power efficiency in relation with growing voltage or luminance is more rapid; likewise, it is seen in *η = f*(*J*) dependence (Figure 5b). In addition, very similar relationships were obtained for several OLEDs produced in subsequent series. However, the observed changes are insignificant, and the results can be considered comparable. On that basis, it can be concluded that the application of the slot-die-coating technique enables the production of OLEDs with parameters close to those produced by the spin-coating method even for multicomponent emissive systems. The beneficial usage of this technique to OLED production has already been reported by other research groups [41,43,49].

### 3.3. OLED Results for Polymer Host–Guest System with TADF

The promising results presented for diodes with a “PVK:PBD + Ir” emissive layer enable further research in this field. Therefore, this technique was also applied to fabricate OLEDs based on the more effective emissive system that was enriched with TADF-type material. Instead of PBD, TADF assistant dopant was employed in the PVK matrix. The application of TADF enables the harvesting of both singlet and triplet excitons, can facilitate proficient energy transfer from the host material to the phosphorescent emitter [66], and finally can improve the OLED efficiency [67]. To meet the basic requirements for efficient energy transfer to the yellowish target emitter [68], as assistant dopant SpiroAC-TRZ with high triplet energy level and blue emission [69] was introduced. Efficient energy transfer between TADF and Ir complex is essential for obtaining good OLED performance. The doping ratio of Ir complex in TADF-enriched matrix should be optimized to acquire high energy transfer efficiency [67,70]. The optimal EML composition of PVK + 25 wt.% SpiroAC-TRZ + 2 wt.% Ir resulted from our previous studies [71].

**Figure 6:**
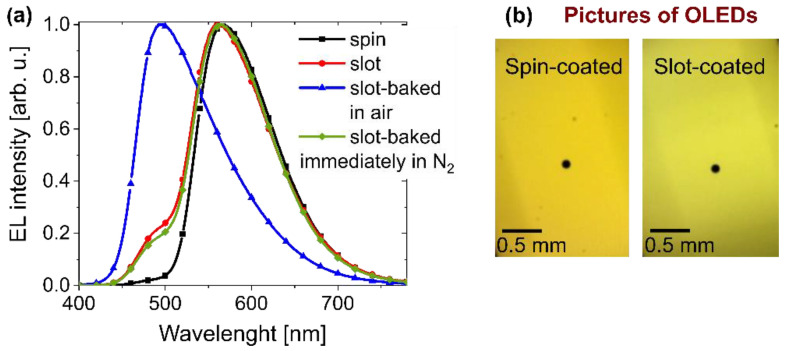
(**a**) Normalized EL spectra and (**b**) pictures of working devices based on emissive layer (PVK + 25 wt.% SpiroAC-TRZ + 2 wt.% Ir) prepared by means of spin- or slot-die coating techniques. Dark dots visible on the pictures are related to the marked measurement areas.

**Figure 7:**
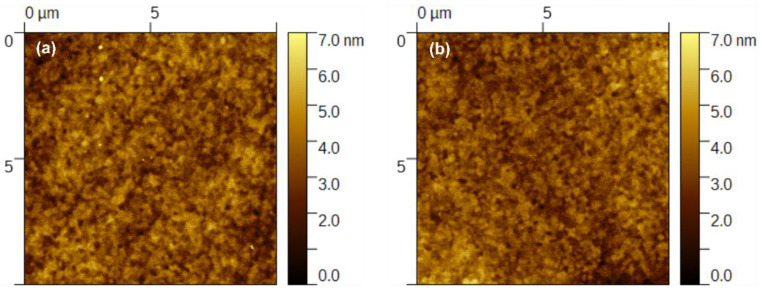
AFM topology of emissive layers (PVK + 25 wt.% SpiroAC-TRZ + 2 wt.% Ir) prepared by means of spin coating (**a**) and slot-die coating (**b**) techniques. Emissive layer deposited on PEDOT:PSS/ITO.

As described in Section 3.2, only EML was deposited by means of the slot-die technique. The process parameters for the deposition of the “PVK-TADF-Ir” layer were generally similar, as for the “PVK:PBD + Ir” layer. However, the solution concentration was changed to 18 mg mL^−1^ and the substrate speed to 10 mm s^−1^. As previously noted, spin- and slot-die-coated layers were baked under a nitrogen atmosphere to remove residual solvent and stabilize the film structure. Samples were observed under a polarizing microscope, and no changes in morphology upon annealing were found. The quality of the slot-die layers was comparable to the ones prepared through the spin-coating technique and good enough for application as EML in OLED. No noticeable differences were observed in the layer properties between the EMLs obtained by those two methods. This can be seen inter alia on the working device pictures (Figure 6b) and on the AFM images (Figure 7).

**Figure 8:**
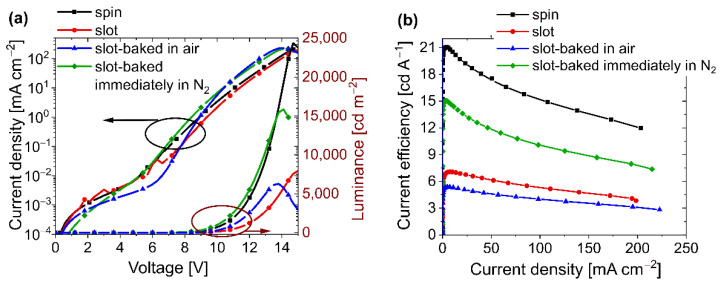
Working parameters of OLEDs based on emissive layer (PVK + 25 wt.% SpiroAC-TRZ + 2 wt.% Ir) prepared by means of spin- or slot-die coating technique. Device structure: ITO/PEDOT:PSS (20 nm)/EML (75 nm)/Bphen (40 nm)/Liq (2 nm)/Ag (100 nm). (**a**) Current density–voltage characteristics (left) and luminance–voltage characteristics (right); (**b**) Current efficiency vs. current density.

However, the method of EML fabrication significantly influences the OLED working parameters (see Figure 6a and Figure 8). In the EL spectrum, an additional band appeared when the EML was fabricated by the novel technique, which is attributed to the TADF emission (490 nm) [69] (Figure 6a, red curve). The observed EL from SpiroAC-TRZ might be related to the incomplete energy transfer from TADF to the Ir-dopant and, consequently, excitation energy loss, resulting in a worse device performance. Furthermore, the *L*–*V* characteristics depicted in Figure 8a revealed a higher turn-on voltage and drastically lower luminance in a similar current-density range. Consequently, a serious decrease in device efficiency by ~3 times was observed in the case of slot-die-coated EML (compare red and black curves). One possible reason for such discrepancies might be a worse morphology of EML obtained by the slot-die technique. However, no differences between spin- and slot-die-coated samples were observed under a microscope and with a profilometer during the coating process optimization. Nonetheless, to exclude the impact of the inferior slot-die-coated layer, the AFM pictures were made (Figure 7). As can be seen in Figure 7, there are no noticeable discrepancies between the EMLs obtained by these two coating techniques. Therefore, the performance loss cannot be assigned to worse layer quality in the case of slot-die coating. Additionally, dark spots were not observed in the working devices (Figure 6b).

In order to clarify the performance loss in the case of slot-die-coated OLEDs, operational lifetime measurements were performed (Figure S7). Normalized EL decay curves of the investigated OLEDs based on spin- and slot-die-coated EML (PVK + 25 wt.% SpiroAC-TRZ + 2 wt.% Ir) were shown as a function of operational time. Two comparison experiments were made with different initial luminance values (*L_0_* = 300 cd m^−2^ and *L_0_* = 1000 cd m^−2^). In both cases, OLEDs with slot-die-coated EML show lower stability and exhibit a faster light intensity reduction at the beginning.

A possible reason for the poorer results, obtained for OLEDs with slot-die-coated EML, might be the presence of trace amounts of oxygen molecules trapped in the bulk of the emissive layer during the deposition process in air. It can be especially expected at the bottom of the thick EML, on the PEDOT:PSS side. The samples annealing in the inert atmosphere and their detainment in the vacuum, before the cathode side deposition, perhaps only release molecules from the near-surface layer, which may be not enough for 75 nm EML. This leads to the assumption that lower device performance and stability might be related to the inferior impact of oxygen traces on TADF material. The disturbance of energy transfer between TADF and Ir complex molecules should be considered [72,73]. Therefore, in the next step, the influence of an air environment was checked to explain the differences in the efficiency of the tested devices.

Previously, the time of the sample exposure to oxygen influence was not controlled and EML was annealed in the glovebox at an unspecified delay after the deposition (results depicted by red curves in Figure 6a and Figure 8). Therefore, to check the oxygen impact, light-emitting layers were prepared in two other ways. One sample was immediately transferred and baked in the glovebox just after the coating process (depicted as green curves in Figure 6a and Figure 8). In the case of the second sample, negative oxygen exposure was enhanced by annealing it in the air (depicted as blue curves in Figure 6a and Figure 8). Afterward, samples were transferred into the evaporation system in order to deposit the following layers in a vacuum. As one can see in Figure 8, the limitation of oxygen impact resulted in a significant improvement of the device’s working parameters (*cfr.* red and green curves). Evidently, the immediate baking process performed in the air contributed to the worsening of the diode parameters (blue lines) mainly by changing the emissive properties of the layer. After extended air exposure, the detected EL spectrum is mostly related to SpiroAC-TRZ emission, and the contribution of the Ir complex electroluminescence is visible only as a long-wavelength tail in the emission band (Figure 6a blue curve). Considering that the fabrication technique generally did not influence the results obtained in the case of PVK:PBD + 2 wt.% Ir layer (see Table 1), it may be suspected that the impact of oxygen on the phosphorescent Ir complex is minimal, due to its low concentration in the layer. This may suggest that oxygen detrimentally affected the energy transfer from the TADF assistant dopant to the phosphorescent emitter [72,73]. This conjecture is especially reasonable in the case of the application of materials utilizing excited triplet states (a TADF material and a phosphorescent emitter) [74]. Disturbance of energy transfer might be related to the quenching of TADF triplet states by oxygen molecules, and consequently, less TADF excitons can be transferred to the Ir complex. It is generally known that oxygen is an effective triplet quencher, so even trace amounts of oxygen molecules that are present throughout the layer could decrease the efficiency of the reverse intersystem crossing process and, as a consequence, the probability of thermally activated delay fluorescence decreases [72]. This process in particular can have a drastic effect on the performance of OLEDs with EML based on PVK + SpiroAC-TRZ + Ir due to the high concentration of TADF material. Furthermore, the formation of exciplex triplet states between TADF and PVK [71] and their subsequent energy transfer to the Ir complex can be disturbed. When such scenarios operate, the quenching of iridium complex emission by oxygen molecules seems to be obvious. The detrimental oxygen impact is also indicated by comparison of lifetime measurements performed for OLEDs, with slot-die and spin-coated EML (see Figure S7). An initial steeper decrease in luminance is observed in the case of slot-die-coated EML, which can also be attributed to the presence of a small amount of oxygen molecules entrapped in the emissive layer.

In summary, even when the influence of oxygen was limited, performing slot-die coating and baking of EML (PVK + 25 wt.% SpiroAC-TRZ + 2 wt.% Ir) in an ambient environment resulted in worse parameters of the device. It is true that the rapid transfer of the layer and its heating in an N_2_ atmosphere contributes to a significant improvement in the efficiency of the diodes. However, the performance is still smaller than that obtained for OLEDs with an emissive layer produced in the glovebox by the spin-coating method (compare the green and black curves in Figure 8b and also the OLED parameters listed in Table 1). Nonetheless, it should be underlined that the maximal current efficiency of OLEDs at a level of 15 cd A^−1^ from multicomponent EML, prepared by the slot-die technique, is a good achievement compared to the results published so far [50]. To our knowledge, it is the second-highest value, just after results obtained by Park et al., who received 29 cd A^−1^ [43]. However, it should be taken into account that in their case, the well-known and the most efficient green iridium complex, tris(2-phenylpyridine)iridium(III), Ir(ppy)_3_, was used as the emitter. For further improvement of the performance of OLEDs obtained by the slot-die coating, the layer printing should be performed in an inert atmosphere to eliminate the negative impact of oxygen. However, it appears that a rapid transfer of freshly printed layers from air to an inert atmosphere and annealing (to minimize the negative oxygen influence) may yield good results and can be implemented for other printing techniques.

## 4. Conclusions

The deposition of multicomponent EMLs by slot-die coating was successfully optimized for OLED application. The obtained results confirmed that the slot-die-coating technique is a suitable method for solution-processed OLEDs. The experiments were carried out on two host–guest systems: PVK:PBD + Ir-dopant and PVK:TADF+ Ir-dopant. The performed studies revealed that controlled deposition of the host–guest systems even based on three components is possible in a way similar to that of the spin-coating method. Appropriate adjustment of the process conditions ensured the desired thickness and smooth topology of the layer. A thorough comparison of the quality of the EMLs obtained by slot-die and spin-coating (microscopy, profilometry, AFM) suggested no impact of the layer topology.

OLEDs fabricated with EML slot-die-coated in air were confronted with those with EML spin-coated in nitrogen. Studies were performed to determine the effect of using this method in industrially acceptable conditions (in air). OLEDs based on PVK:PBD + 2 wt.% Ir-dopant had comparable performances (~13 cd A^−1^) when using the slot-die as well as spin-coating technique. When the EML system was enriched with the TADF assistant dopant, the performance of OLEDs was enhanced. OLEDs spin-coated in a nitrogen atmosphere, based on PVK with 25 wt.% SpiroAC-TRZ and 2 wt.% Ir-dopant, showed high current efficiency (21 cd A^−1^). Nevertheless, devices with the same EML system that was slot-die-coated in air exhibited approximately three-times lower efficiency.

Analysis of the EL spectra and OLED working parameters suggested a detrimental oxygen impact in the case of slot-die coating. The observed significant decrease in efficiency is caused by ambient deposition conditions for the slot-die coating. Typically, oxygen molecules trapped in part of the surface layer can be easily removed by the annealing process in inert conditions. Accordingly, this method can be successfully applied for host–guest systems with a small phosphorescent emitter concentration. However, in the case of the relatively thick layers (~70 nm), the presence of some trapped oxygen molecules cannot be excluded. In systems with a high contribution of materials utilizing triplet states, such as in the case of the system with TADF assistant dopant, even a trace amount of oxygen has a significant adverse effect on the working OLED. Oxygen traces might decrease the efficiency of the reverse intersystem crossing process in TADF and can additionally disturb the formation of exciplex triplet states between matrix components. All of these processes result in a reduction in device performance.

The elaborated post-treatment approach enabled doubling of the efficiency of OLEDs with EML based on PVK with 25 wt.% SpiroAC-TRZ and 2 wt.% Ir-dopant. The devices exhibited maximal current efficiency at a level of 15 cd A^−1^, which is a fairly good achievement compared to the published results employing the slot-die-coating method. Furthermore, to the best of our knowledge, this is the first time that the slot-die technique has been successfully used to produce an emissive layer enriched with a TADF assistant dopant. Immediate annealing in the inert atmosphere of the freshly coated layers is a good hint for future research. This approach may be easily implemented to other printing techniques and can contribute to performance improvement.

## Figures and Tables

**Table 1:** OLED parameters.

Preparation Conditions	*EL*_max_ (nm)	*L*_max_ (cd m^−2^)	*η*_max_ (cd A^−1^)
**EML: PVK:PBD + 2 wt.% Ir**			
Spin-coated and baked in N_2_	558	11,500	12.8
Slot-die-coated in air and baked in N_2_ with no controlled delay	558	12,000	13.8
**EML: PVK + 25 wt.% SpiroAC-TRZ + 2 wt.% Ir**			
Spin-coated and baked in N_2_	560	7,900	7.1
Slot-die-coated in air and baked in N_2_ with no controlled delay	495	6,300	5.4
Slot-die-coated and baked in air	556	15,900	15.0
Slot-die-coated in air and baked immediately in N_2_	566	24,300	21.0

## Data Availability

Not applicable.

## Supplementary Materials

The following supporting information can be downloaded at: https://www.mdpi.com/article/10.3390/polym14163363/s1, Figure S1: Thickness of EML films including PEDOT:PSS, where EML (PVK:PBD + 2 wt.% Ir) was deposited through a slot-die coating at different process parameters. The nozzle-substrate distance was kept constant at 100 μm. The EML components were dissolved in chlorobenzene with 21 mg mL^−1^ concentration. The vertical bars indicate the range of variation of the films thickness in different places of the same sample.; Figure S2. Optical microscope picture of emissive layer (PVK:PBD + 2 wt.% Ir) deposited by slot-die coating from chlorobenzene. The process parameters: temperature: 45 °C, nozzle-substrate distance: 100 μm, flow rate: 5 μL min^−1^, substrate speed: 0.2 mm s^−1^. Emissive layer deposited on PEDOT:PSS. Visible vertical line is related to ITO edge. EML/PEDOT:PSS/ITO/glass on the right and EML/PEDOT:PSS/glass on the left side of the picture.; Figure S3. Optical microscope picture of emissive layer (PVK:PBD + Ir) deposited by slot-die coating technique from anisole. Fixed process conditions: substrate speed = 0.3 mm s^−1^, *T* = 35°C, perpendicular blade, distance nozzle-substrate: 100 μm, solution flow rate = 10 μL min^−1^. Emissive layer deposited on PEDOT:PSS. EML/PEDOT:PSS/ITO/glass (the right substrate side), EML/PEDOT:PSS/glass (left).; Figure S4. Optical microscope pictures of emissive layer (PVK:PBD + Ir) deposited by slot-die coating technique from different solvents. Fixed process conditions: substrate speed: 0.3 mm s^−1^, *T* = 40–45 °C, perpendicular blade, distance nozzle-substrate: 100 μm, solution flow rate: 5–7 μL min^−1^. Emissive layer deposited on PEDOT:PSS. EML/PEDOT:PSS/ITO/glass (the right substrate side), EML/PEDOT:PSS/glass (left).; Figure S5. Two optical microscope pictures of emissive layer (PVK:PBD + Ir) deposited by slot-die coating technique. Emissive layer deposited on PEDOT:PSS. EML/PEDOT:PSS/ITO/glass (the right substrate side), EML/PEDOT:PSS/glass (left). Figure S6. Two optical microscope pictures of emissive layer (PVK:PBD + Ir) deposited by spin coating technique. Emissive layer deposited on PEDOT:PSS. EML/PEDOT:PSS/ITO/glass (the right substrate side), EML/PEDOT:PSS/glass (left).; Figure S7. Normalized EL decay curves of the investigated OLEDs based on spin- and slot-die-coated EML (PVK + 25 wt.% SpiroAC-TRZ + 2 wt.% Ir) as a function of operational time (at initial luminance *L*_0_ = 300 cd m^−2^ and *L*_0_ = 1000 cd m^−2^); measured in the constant current regime.
