# Supplementary material for: Efficient OLEDs Based on Slot-Die-Coated Multicomponent Emissive Layer
Source: Polymers (Basel). 2022 Aug 17;14(16):3363. doi: 10.3390/polym14163363 (PMC9413595; doi:10.3390/polym14163363)
Supplement: Supplementary file 1 [file polymers-14-03363-s001.zip › polymers-1791194-supplementary.pdf]

Supplementary Materials

Efficient OLEDs Based on Slot-Die-Coated Multicomponent Emissive Layer

Ewelina Witkowska, Ireneusz Glowacki, Tung-Huei Ke, Pawel Malinowski, Paul Heremans*

Process parameters adjustments were made for emission layer (EML) consisted of matrix mixture of poli(*N*-vinylcarbazole) (PVK) with oxadiazole derivative (PBD) with addition of 2 wt% of [bis(benzo[*h*]quinolinato-*N*,$C^{10\prime}$){4-((1-naphtyl)imino)-pent-2-en-2-olato-*N*,*O*} iridium(III)] iridium complex (Ir). After EML coating, all samples were baked under a nitrogen atmosphere in order to remove the residual amounts of the solvent and stabilize the film structure. It is known that the ability to form the layer, with a certain thickness and proper quality, is influenced not only by the method parameters but also by the energy of the surface tension. For this reason, optimization of the EML slot-die coating was carried out directly on substrates with a previously spin-coated poly(3,4–ethylenedioxythiophene) and poly(styrene sulfonate) mixture (PEDOT:PSS) film (20-25 nm), that was introduced as the hole injection layer (HIL). After coating and drying, the film morphology was examined using profilometry and under an optical microscope. In solution-processable OLEDs, choosing a proper process conditions is necessary for uniform coating, but simultaneously the underlayer cannot be dissolved or swelled. Therefore, all given thicknesses refer to the EML together with applied HIL.

The first trials were performed for chlorobenzene, because it was used in previously optimized spin coating process. Thickness dependence on most crucial process parameters (substrate speed, solution flow rate and temperature) is presented in Figure S1. Nonetheless, the film quality was disappointing due to the inhomogeneous thickness and quality of the layer. As can be seen in Figure S2, the layer was folded and stripes across the substrate movement were observed. It is commonly known that not only deposition parameters impact the layer

properties, but solvent selection is also crucial, due to the possibility to change the surface tension. Therefore, in the next step different solvents were investigated (Figure S3 and S4).

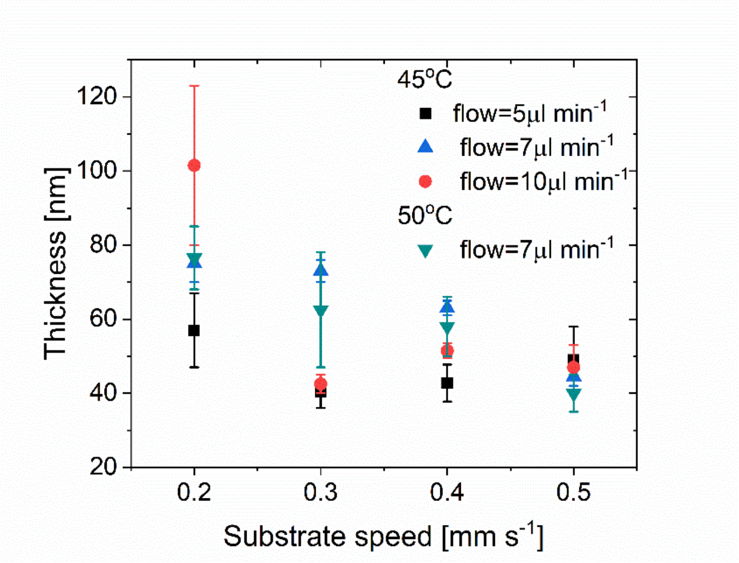

Figure S1. Thickness of EML films including PEDOT:PSS, where EML (PVK:PBD + 2 wt.% Ir) was deposited through a slot-die coating at different process parameters. The nozzle-substrate distance was kept constant at 100 µm. The EML components were dissolved in chlorobenzene with 21 mg mL^{-1} concentration. The vertical bars indicate the range of variation of the films thickness in different places of the same sample.

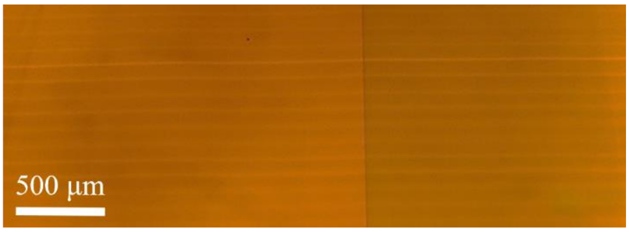

Figure S2. Optical microscope picture of emissive layer (PVK:PBD + 2 wt.% Ir) deposited by slot-die coating from chlorobenzene. The process parameters: temperature: 45ºC, nozzle-substrate distance: 100 µm, flow rate: 5 µL min^{-1}, substrate speed: 0.2 mm s^{-1}. Emissive layer deposited on PEDOT:PSS. Visible vertical line is related to ITO edge. EML/PEDOT:PSS/ITO/glass on the right and EML/PEDOT:PSS/glass on the left side of the picture.

Thorough process parameters adjustments, seen in Figure 2 of the main manuscript, were made for anisole. Microscope images showed good layer quality (Figure S3). Similar images were received for fixed process condition in the following range: flow rate = 5-25 µL min^{-1}, T = 35-50ºC, substrate speed = 0.1-0.7 mm s^{-1}. Nonetheless, when morphology

was good the thickness of emissive layer together with PEDOT:PSS was only ~40 nm, that is not enough for application in such OLEDs structure (see Figure 6a) for their stable work. Therefore, different solvents were introduced to the studies (Figure S4).

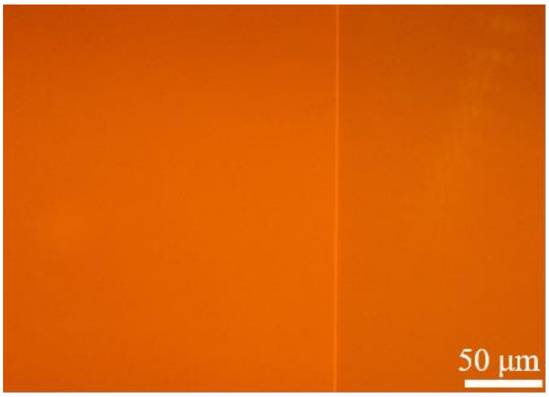

Figure S3. Optical microscope picture of emissive layer (PVK:PBD+Ir) deposited by slot-die coating technique from anisole. Fixed process conditions: substrate speed = 0.3 mm s^{-1}, T = 35°C, perpendicular blade, distance nozzle-substrate: 100 μm, solution flow rate =10 μL min^{-1}. Emissive layer deposited on PEDOT:PSS. EML/PEDOT:PSS/ITO/glass (the right substrate side), EML/PEDOT:PSS/glass (left).

Comparison of the layers received from different solvents:

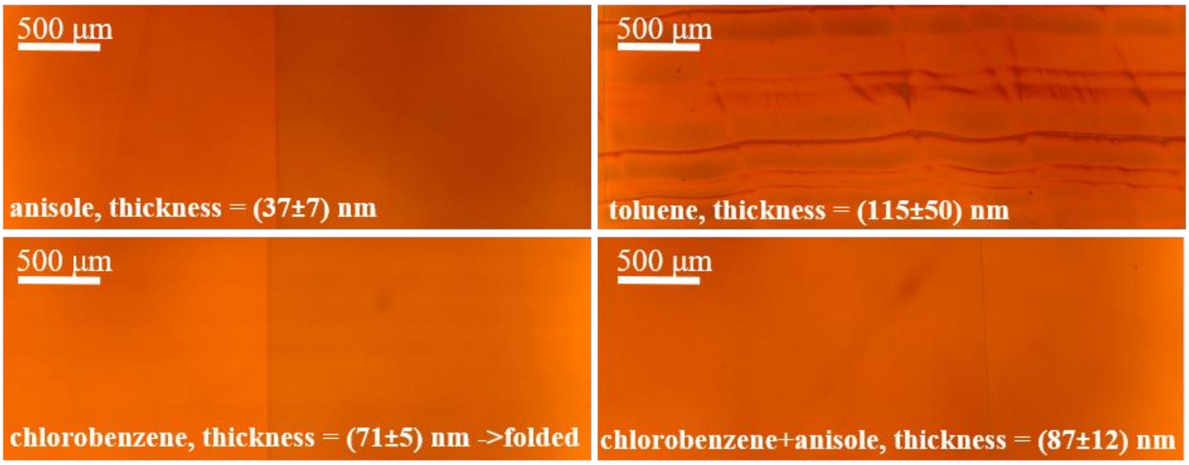

Figure S4. Optical microscope pictures of emissive layer (PVK:PBD+Ir) deposited by slot-die coating technique from different solvents. Fixed process conditions: substrate speed: 0.3 mm s^{-1}, T = 40-45°C, perpendicular blade, distance nozzle-substrate: 100 μm, solution flow rate: 5-7 μL min^{-1}. Emissive layer deposited on PEDOT:PSS. EML/PEDOT:PSS/ITO/glass (the right substrate side), EML/PEDOT:PSS/glass (left).

Optimized parameters:

In order to receive 75 nm emissive layer mixture of anisole and chlorobenzene (1:1 by volume) with 21 mg mL^{-1} material concentration was applied. Following parameters were finally chosen: nozzle under angle (~35º), substrate temperature: 40ºC, distance between the nozzle and the substrate: 50 μm. In order to create the meniscus, 20 μL was pumped with solution flow speed of 50 μL min^{-1}, whereas during the deposition, solution flow was reduced to 10 μL min^{-1} and substrate was moving at 7.0 mm s^{-1}. Comparative layer quality was obtained as for EML obtained by spin coating technique (compare Figure S5 and S6).

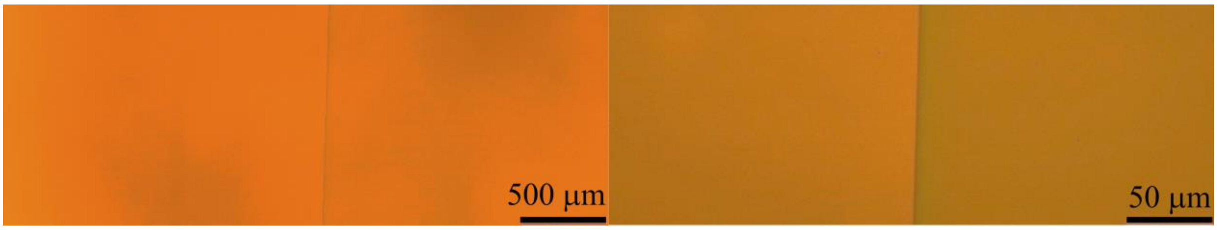

Figure S5. Two optical microscope pictures of emissive layer (PVK:PBD+Ir) deposited by slot-die coating technique. Emissive layer deposited on PEDOT:PSS. EML/PEDOT:PSS/ITO/glass (the right substrate side), EML/PEDOT:PSS/glass (left).

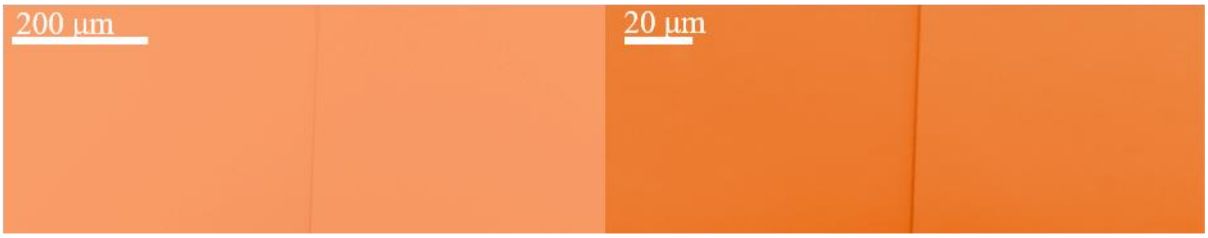

Figure S6. Two optical microscope pictures of emissive layer (PVK:PBD+Ir) deposited by spin coating technique. Emissive layer deposited on PEDOT:PSS. EML/PEDOT:PSS/ITO/glass (the right substrate side), EML/PEDOT:PSS/glass (left).

OLED results for polymer host-guest system with TADF

In order to to clarify the performance loss, observed for slot-die coated OLEDs, operational lifetime measurements were conducted.

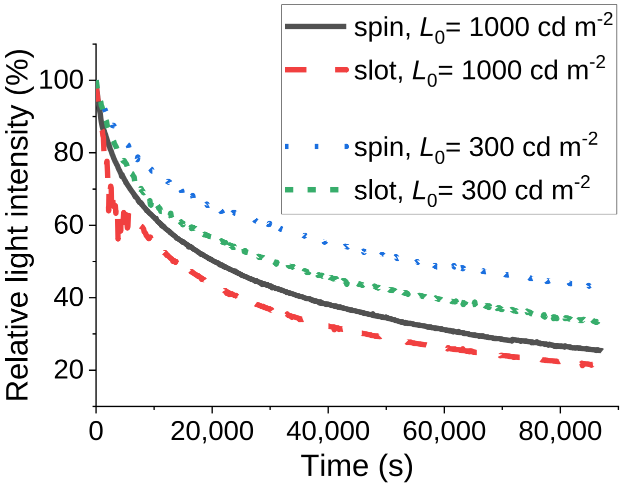

Figure S7. Normalized EL decay curves of the investigated OLEDs based on spin- and slot-die coated EML (PVK+25 wt.% SpiroAC-TRZ+2 wt.% Ir) as a function of operational time (at initial luminance L_0 = 300 cd m^{-2} and L_0 = 1000 cd m^{-2}); measured in the constant current regime.
